# Are the genomes of motile Aeromonas species from the aquatic environment shaped by local geography and host species?

**DOI:** 10.1099/mgen.0.001618

**Published:** 2026-04-24

**Authors:** Christopher J. Payne, Vo Hong Phuong, Heri Kurniawan, Francis S. Legario, Le Hong Phuoc, Margaret Crumlish

**Affiliations:** 1 Institute of Aquaculture, Faculty of Natural Sciences, University of Stirling, Stirling, UK; 2 Southern Monitoring Center for Aquaculture Environment and Epidemic, Research Institute for Aquaculture No. 2, Ho Chi Minh City, Vietnam; 3 The Agency for Control and Supervision of Marine and Fisheries Products, the Ministry of Marine Affairs and Fisheries of Indonesia, Jakarta, Indonesia; 4 Biotech & Microbiology Laboratories/Natural Sciences Department, Iloilo Science and Technology University, Iloilo City, 5000, Philippines

**Keywords:** * Aeromonas*, antimicrobial resistance genes, aquaculture, comparative genomics, virulence factors

## Abstract

*Aeromonas dhakensis* and *Aeromonas hydrophila* cause significant economic losses within the global aquaculture sector, affecting numerous farmed fish species and posing a zoonotic threat to human health. In this study, we sequenced, assembled and analysed the genomes of seven and five *A. dhakensis* and *A. hydrophila* isolates, respectively, recovered from disease outbreaks in various fish hosts across Southeast Asia using a hybrid sequencing approach with Illumina and Oxford Nanopore technologies. To assess the relatedness of our isolates with those from the global aquatic environment, with particular reference to aquaculture-relevant systems, and compare their resistome and virulome profiles, we also conducted comparative genomic analysis with an additional 57 publicly available genomes of *A. dhakensis* and *A. hydrophila*, recovered from different aquatic sources. Findings from this study revealed large genomic variability across global *Aeromonas* populations, with a clear distinction in the pan-genome between *Aeromonas* species. *In silico* multi-locus sequence type analysis revealed a wide distribution of sequence types (STs) 656 and 251 in Asia in *A. dhakensis* and *A. hydrophila*, respectively, although the novel STs detected in Indonesia and the Philippines suggest local adaptation of populations circulating in these countries. Analysis of mobile genetic elements revealed plasmids, insertion sequences and genomic islands to be country- or host-specific. Exploration of resistome data revealed a high prevalence of multidrug resistance across Southeast Asia, with genomes frequently carrying resistance genes against antibiotic classes commonly used across the aquaculture sector, including potentiated sulphonamides and tetracyclines. However, several antimicrobial resistance genes were found to be country- or host-specific, suggesting local adaptation to anthropogenic or environmental conditions within specific regions. A diverse repertoire of virulence genes was also detected in this study, with *A. hydrophila* demonstrating greater diversity in virulence genes compared with *A. dhakensis*. Country-specific virulence pathways were also noted for several toxins and secretion systems, including *aerA/act*, *rtx* and type III and IV secretion systems. This work provides new insights into the genomic features and relatedness of *Aeromonas* spp. circulating within aquaculture systems in Southeast Asia. Further, findings from comparative genomic analysis highlight the influence of geographical or host pressures on the molecular drivers of antimicrobial resistance and pathogenicity, directly impacting animal health and aquaculture production.

**Impact Statement**

Motile *Aeromonas* species, including *Aeromonas dhakensis* and *Aeromonas hydrophila*, are economically important bacterial pathogens that continue to post a significant threat to the production of freshwater fish across the global aquaculture sector. In this study, we sequenced the genomes of seven and five *A. dhakensis* and *A. hydrophila* recovered from different fish species across Southeast Asia. We then performed comparative genomic analysis with publicly available motile *Aeromonas* genomes from different aquatic sources, investigating the *Aeromonas* pan-genome and genomic drivers behind antimicrobial resistance and pathogenicity. We showed that sequence types (STs) 656 and 251 dominated the genome collection of *A. dhakensis* and *A. hydrophila* from Vietnam, respectively, although novel STs were detected in Indonesia and the Philippines. We also demonstrated high levels of predicted antibiotic resistance across Southeast Asia, with several antibiotic resistance genes against popular antibiotic compounds detected. Further, both country- and host-specific resistome profiles were identified. A diverse virulome was detected in both *A. dhakensis* and *A. hydrophila*, albeit shaped by geography. This work has considerably expanded our understanding of the genomic features and relatedness of *Aeromonas* spp. circulating within global aquaculture systems. Findings from this study will contribute to ongoing surveillance programmes and help to inform disease control and management strategies applied across the Southeast Asia aquaculture sector.

## Data Summary

Whole-genome sequencing data generated in the study are available in the National Center for Biotechnology Information (NCBI) Sequence Read Archive repository under the accession number PRJNA1101888, and the accession numbers for all datasets used are provided in Table 1. The authors confirm that all supporting data, code and protocols have been provided within the article or through supplementary data files.

## Introduction

The genus *Aeromonas* (phylum, *Proteobacteria*; class, *Gammaproteobacteria*; order, *Aeromonadales*, and family, *Aeromonadaceae*) is characterized as Gram-negative, rod-shaped bacteria [1]. The genus is found ubiquitously in the aquatic environment, primarily inhabiting freshwater sources, although representative isolates have also been recovered from marine environments as well as potable and wastewater sources [2,3]. First recovered in the late nineteenth century, where isolates were identified as *Bacillus hydrophilus fuscus* but re-classified as *Aeromonas hydrophila* in 1943, the genus now comprises 34 valid species [4]. Several *Aeromonas* species cause disease in humans and terrestrial animals [5,9]. Likewise, members of this genus also infect a range of aquatic animals, including fish, marine reptiles and marine mammals [10,12].

In aquaculture, several *Aeromonas* species are known to cause high mortality and significant economic losses in production systems through disease outbreaks from furunculosis and motile *Aeromonas* septicaemia (MAS), caused by *Aeromonas salmonicida* and members of the motile *Aeromonas* species, respectively [13]. The close relatedness of the motile *Aeromonas* species associated with natural disease outbreaks in farmed fish has led to the description ‘motile *Aeromonas* complex’, which generally presents externally in infected fish as reddened or eroded fins, ulceration, scale loss and internally as haemorrhaging and swelling of the kidney and spleen, although host-specific differences have been noted [14]. Taxonomic assignment of motile *Aeromonas* species has been historically challenging, with *Aeromonas dhakensis* isolates often misidentified as *A. hydrophila* due to similarities in phenotypic profiles between the two species or the poor discriminatory power of traditional housekeeping genes and databases used in species identification [15]. However, advancement in molecular approaches, including whole-genome sequencing and average nucleotide identity (ANI), has greatly improved species-level identification, particularly in distinguishing *A. dhakensis* and *A. hydrophila* [14]. As such, whilst *A. hydrophila* is frequently associated with MAS outbreaks in several fish groups, including carps, tilapia and catfish, *A. dhakensis* is now considered an emerging bacterial pathogen of importance within the aquaculture sector [16].

Advances in next-generation sequencing have revolutionized the study of bacterial genomes, leading to deeper insights into genome structure and evolution, as well as identifying genetic drivers in pathogenicity and antimicrobial resistance (AMR). Indeed, comparative genomics has allowed for a comprehensive understanding of the genome structure and variability in several *Aeromonas* species, including *Aeromonas caviae*, *A. salmonicida* and *Aeromonas veronii* [17,20]. Comparative genomics has also revealed country- or host-specific genotypes in other notable bacterial pathogens affecting fish [21,23]. In this study, the genomes of seven *A. dhakensis* and five *A. hydrophila* isolates from various hosts in Indonesia, the Philippines and Vietnam were sequenced using Illumina and Oxford Nanopore Technology platforms. To better understand the population dynamics of *A. dhakensis* and *A. hydrophila* circulating the global aquatic environment, comparative genomic analysis was conducted among these and an additional 57 publicly available genomes associated with different aquatic hosts (including aquaculture-relevant species) and geographical locations, to determine their phylogenetic relatedness and provide deeper insights into unique genomic factors related to antimicrobial resistance, evolution and virulence.

## Methods

### *Aeromonas* isolates

A total of 69 motile *Aeromonas* isolates (*n*=26 *A*. *dhakensis* and *n*=43 *A*. *hydrophila*) from various aquatic hosts and different geographical locations were investigated in this study. The genome sequences from 19 and 38 *A*. *dhakensis* and *A. hydrophila* isolates, respectively, were retrieved from the National Center for Biotechnology Information (NCBI) RefSeq database (28 August 2024, Table 1). Genomes were selected based on the following criteria: where ‘Complete Genome’ or ‘Chromosome’ level was available, and the NCBI taxonomy check verified species identity as either *A. dhakensis* or *A. hydrophila*. The genomes of the other 12 isolates (*n*=7 *A*. *dhakensis* and *n*=5 *A. hydrophila*) were sequenced in this study (Table 1).

**Table 1.** Genomic features of the *A. dhakensis* and *A. hydrophila* isolates investigated in this study

Isolate	Accession	Species	Country	Host	Completeness (%)	Contamination (%)	Size (Mbp)	GC %	CDS (total)	rRNA gene	No. plasmid (accession)
Newly sequenced genomes											
AD-92HK1	SAMN41005112	*A. dhakensis*	Indonesia	*Oreochromis niloticus*	100	0	4.83	61.64	4258	31	–
AD-69	SAMN41005113	*A. dhakensis*	The Philippines	*Oreochromis niloticus*	100	0	4.89	61.56	4367	31	–
AD-VN1	SAMN41005116	*A. dhakensis*	Vietnam	*Channa striata*	100	0	4.97	61.1	4411	31	4 (CP152260, CP152261, CP152262,CP152263)
AD-VN2	SAMN41005117	*A. dhakensis*	Vietnam	*Channa striata*	100	0	4.96	61.11	4403	31	4 (CP152255,CP152256, CP152257, CP152258)
AD-27	SAMN41005114	*A. dhakensis*	Vietnam	*Pangasianodon hypophthalmus*	100	0	4.94	61.22	4401	31	4 (CP152270, CP152271, CP152272, CP152273)
AD-1	SAMN41005115	*A. dhakensis*	Vietnam	*Pangasianodon hypophthalmus*	100	0	4.97	60.97	4431	31	4 (CP152265, CP152266, CP152267, CP152268)
AD-9	SAMN41005118	*A. dhakensis*	Vietnam	*Pangasianodon hypophthalmus*	100	0	5.03	61.01	4497	31	4 (CP152250, CP152251, CP152252, CP152253)
AH-40	SAMN41005119	*A. hydrophila*	Vietnam	*Pangasianodon hypophthalmus*	100	0	4.84	61.01	4294	31	1 (CP152248)
AH-32	SAMN41005121	*A. hydrophila*	Vietnam	*Pangasianodon hypophthalmus*	100	0	5.03	60.91	4478	31	2 (CP152242, CP152243)
AH-34	SAMN41005120	*A. hydrophila*	Vietnam	*Pangasianodon hypophthalmus*	100	0	5.07	60.83	4521	31	2 (CP152245, CP152246)
AH-12	SAMN41005122	*A. hydrophila*	Vietnam	*Pangasianodon hypophthalmus*	100	0	5.06	60.85	4501	31	2 (CP152239, CP152240)
AH-7	SAMN41005123	*A. hydrophila*	Vietnam	*Pangasianodon hypophthalmus*	100	0	5.1	60.81	4536	31	1 (CP152237)
RefSeq genomes from NCBI											
Aer_OnIF1	GCF_022703095.1	*A. dhakensis*	Brazil	*Oreochromis niloticus*	99.91	0.23	4.83	61.65	4290	31	–
Aer_On24M	GCF_017310095.1	*A. dhakensis*	Brazil	*Oreochromis niloticus*	99.73	0.29	4.93	61.37	4410	31	–
Aer_Pi12.1HTAS	GCF_025266835.1	*A. dhakensis*	Brazil	*Phractocephalus hemioliopterus*	99.95	0.61	4.82	61.35	4303	25	1 (CP050696)
202108B1	GCF_034143565.1	*A. dhakensis*	China	*Ancherythroculter nigrocauda*	99.96	0.23	5.04	60.97	4457	31	4 (CP133574, CP133575, CP133576, CP133577)
202108 C2	GCF_035658375.1	*A. dhakensis*	China	*Ctenopharyngodon idella*	99.96	0.23	5	61.03	4409	31	–
b2-100	GCF_023920205.1	*A. dhakensis*	China	*Danio rerio*	99.92	0.56	5.02	61.46	4537	31	–
VA54	GCF_037076385.1	*A. dhakensis*	Vietnam	*Anabas testudineus*	99.96	0.51	4.85	61.55	4274	31	–
VA6	GCF_037101205.1	*A. dhakensis*	Vietnam	*Mastacembelus*	99.93	0.58	4.97	61.43	4379	31	1 (AP027932)
TN45	GCF_905132775.1	*A. dhakensis*	Vietnam	*Pangasianodon hypophthalmus*	99.96	0.2	4.77	61.4	4318	2	4 (LR963080, LR963081, LR963082, LR963083)
TN49	GCF_905132875.1	*A. dhakensis*	Vietnam	*Pangasianodon hypophthalmus*	99.96	0.31	4.83	61.32	4382	2	4 (LR963108, LR963109, LR963110, LR963111)
TN50	GCF_905132865.1	*A. dhakensis*	Vietnam	*Pangasianodon hypophthalmus*	99.96	0.31	4.83	61.31	4386	3	4 (LR963095, LR963096, LR963097, LR963098)
TN59	*GCF_905132855.1*	*A. dhakensis*	Vietnam	*Pangasianodon hypophthalmus*	99.96	0.31	4.83	61.31	4386	3	4 (LR963085, LR963086, LR963087, LR963088)
TN10	GCF_905132905.1	*A. dhakensis*	Vietnam	*Pangasianodon hypophthalmus*	99.96	0.31	4.83	61.32	4383	2	4 (LR963100, LR963101, LR963102, LR963103)
TN11	GCF_905132845.1	*A. dhakensis*	Vietnam	*Pangasianodon hypophthalmus*	99.96	0.31	4.83	61.32	4384	2	4 (LR963090, LR963091, LR963092, LR963093)
TN4	GCF_905132895.1	*A. dhakensis*	Vietnam	*Pangasianodon hypophthalmus*	99.96	0.31	4.83	61.32	4381	2	4 (LR963123, LR963124, LR963125, LR963126)
TN14	GCF_905132925.1	*A. dhakensis*	Vietnam	*Pangasianodon hypophthalmus*	99.96	0.31	4.82	61.34	4367	1	2 (LR963105, LR963106)
TN3	GCF_905132935.1	*A. dhakensis*	Vietnam	*Pangasianodon hypophthalmus*	99.96	0.31	4.83	61.32	4381	3	4 (LR963113, LR963114, LR963115, LR963116)
TN5	GCF_905132915.1	*A. dhakensis*	Vietnam	*Pangasianodon hypophthalmus*	99.96	0.31	5.83	61.32	4383	3	4 (LR963118, LR963119, LR963120, LR963121)
VA84	GCF_037076415.1	*A. dhakensis*	Vietnam	*Pangasius bocourti*	99.96	0.51	4.85	61.55	4275	31	–
T4	GCF_905132965.1	*A. hydrophila*	Bangladesh	*Labeo rohita*	98.14	1.19	4.82	61.21	4431	1	–
Aer_Brac14A	GCF_022700855.1	*A. hydrophila*	Brazil	*Dendrocephalus brasiliensis*	98.25	0.96	4.76	61.47	4224	28	–
Aer_Brac66	GCF_022700815.1	*A. hydrophila*	Brazil	*Dendrocephalus brasiliensis*	98.25	0.96	4.75	61.47	4220	27	–
Brac6	GCF_017310195.1	*A. hydrophila*	Brazil	*Dendrocephalus brasiliensis*	98.25	0.96	4.77	61.46	4225	31	–
Aer_LaG33	GCF_017310135.1	*A. hydrophila*	Brazil	*Lophiosilurus alexandri*	98.25	0.96	4.75	61.47	4225	28	–
Aer_LaG34	GCF_017310075.1	*A. hydrophila*	Brazil	*Lophiosilurus alexandri*	97.93	1.28	4.72	61.54	4224	9	–
Aer_OnP2.2	GCF_017310155.1	*A. hydrophila*	Brazil	*Oreochromis niloticus*	98.25	1.28	4.75	61.49	4224	22	–
Aer_OnP4.2	GCF_017310115.1	*A. hydrophila*	Brazil	*Oreochromis niloticus*	97.93	1.28	4.75	61.49	4237	24	–
OnP3.1	GCF_017310215.1	*A. hydrophila*	Brazil	*Oreochromis niloticus*	98.25	0.96	4.77	61.46	4224	31	–
Aer_Pi25.1HTAS	GCF_022700835.1	*A. hydrophila*	Brazil	*Phractocephalus hemioliopterus*	98.58	1.64	4.81	61.23	4302	28	–
516 (Sample ID B3)	GCF_028621795.1	*A. hydrophila*	China	Aquatic animal	97.71	3.3	5.02	60.79	4472	31	–
516 (Sample ID HG13)	GCF_029320915.1	*A. hydrophila*	China	Aquatic animal	96.93	3.97	5.02	60.76	4521	31	1 (CP119765)
ZYAH72	GCF_003491225.1	*A. hydrophila*	China	*Carassius carassius*	98.82	0.82	5.16	60.68	4618	31	–
NJ-35	GCF_001019645.1	*A. hydrophila*	China	*Carassius carassius*	98.19	1.47	5.28	60.51	4776	31	–
JBN2301	GCF_001455365.1	*A. hydrophila*	China	*Carassius carassius*	98.81	1.22	5.13	60.41	4640	31	3 (CP013179, CP013180, CP013181)
J-1	GCF_000819505.1	*A. hydrophila*	China	*Carassius carassius*	97.55	1.14	5	60.89	4481	31	–
AH10	GCF_000963645.1	*A. hydrophila*	China	*Ctenopharyngodon idella*	97.28	0.83	4.91	61.15	4386	33	–
D4	GCF_001518775.1	*A. hydrophila*	China	Fish	98.33	1.98	5.1	60.46	4770	31	4 (CP013966, CP013967, CP013968, CP013969)
AH230115	GCF_037765365.1	*A. hydrophila*	China	*Hypophthalmichthys nobilis*	98.93	1.43	5.27	60.48	4688	31	2 (CP148750, CP148751)
Ah27	GCF_023823085.1	*A. hydrophila*	China	*Ictalurus punctatus*	97.81	0.82	5.1	60.76	4576	31	–
HX-3	GCF_009791455.1	*A. hydrophila*	China	*Larimichthys crocea*	98.03	1.03	4.94	61	4376	31	–
LHW39	GCF_011602425.1	*A. hydrophila*	China	*Megalobrama amblycephala*	98	1.22	5.1	60.8	4583	31	–
MaAh001	GCF_035319865.1	*A. hydrophila*	China	*Monopterus albus*	98.38	0.81	4.89	61.2	4318	31	–
GYK1	GCF_001683535.1	*A. hydrophila*	China	*Siniperca chuatsi*	98.11	1.14	4.95	60.82	4391	31	–
AC185	GCF_022631175.1	*A. hydrophila*	South Korea	*Anguilla rostrata*	99.3	1.78	4.96	61.34	4422	31	–
AC133	GCF_022631195.1	*A. hydrophila*	South Korea	*Carassius carassius*	97.51	0.82	5.04	60.9	4492	31	–
LP0103	GCF_022557195.1	*A. hydrophila*	Taiwan	*Hypostomus plecostomus*	97.63	1.26	5.02	60.91	4437	31	–
AL06-06	GCF_000940915.1	*A. hydrophila*	USA	*Carassius auratus*	98.01	2.34	4.88	61.36	4450	31	3 (CP010948, CP01949, CP01950)
PC-104A	GCF_000635955.1	*A. hydrophila*	USA	Catfish	99.07	0.82	5.02	60.82	4515	31	–
3019	GCF_018802385.1	*A. hydrophila*	USA	Fish	96.73	3.94	5.13	60.95	4687	31	–
AL09-71	GCF_000633175.1	*A. hydrophila*	USA	*Ictalurus punctatus*	99.11	0.82	5.02	60.82	4511	31	–
ML09-119	GCF_000401555.1	*A. hydrophila*	USA	*Ictalurus punctatus*	99.42	0.82	5.02	60.82	4504	31	–
AG-2013-AG1	GCF_029027865.1	*A. hydrophila*	Vietnam	Catfish	98.51	1.09	4.94	60.93	4440	2	–
TN42	GCF_905132945.1	*A. hydrophila*	Vietnam	*Pangasianodon hypophthalmus*	97.98	1.33	4.95	60.95	4478	3	2 (LR963130, LR963131)
TN46	GCF_905132995.1	*A. hydrophila*	Vietnam	*Pangasianodon hypophthalmus*	97.98	1.33	4.95	60.95	4472	3	2 (LR963134. LR963133)
TN22	GCF_905132975.1	*A. hydrophila*	Vietnam	*Pangasianodon hypophthalmus*	97.98	1.33	4.96	60.93	4480	4	2 (LR963136, LR963137)
TN27	GCF_905132955.1	*A. hydrophila*	Vietnam	*Pangasianodon hypophthalmus*	97.98	1.33	4.95	60.97	4471	3	1 (LR963128)
TN1	GCF_905132985.1	*A. hydrophila*	Vietnam	*Pangasianodon hypophthalmus*	98.51	1.14	4.94	60.91	4456	3	2 (LR963139, LR963140)

CDS, coding sequence.

The 12 *Aeromonas* isolates sequenced in this study were recovered from storage on Protect Beads (SLS, UK) at −80 °C and grown on tryptone soya agar (E and O Laboratories Ltd., UK) at 28 °C for 24 h. All bacterial isolates were grown in 10 ml tryptone soya broth (Oxoid, UK) for 18 h at 28 °C, under agitation (140 r.p.m.). High molecular weight DNA was extracted from 1 ml of each bacterial culture using the Wizard^®^ HMW DNA Extraction kit (Promega, UK) following the manufacturer’s instructions. A minor exception was the addition of 5 µl of RNase A solution to the cell lysate, and DNA was eluted in 85 µl of EB buffer (Qiagen, UK). Before purification, extracted DNA was treated with RNAse A/T1 (20 µl ml^−1^) (Fisher Scientific, UK) and incubated at 37 °C for 30 min. Following additional RNAse treatment, DNA was further purified using the DNeasy PowerClean Pro kit (QIAGEN, UK) following the manufacturer’s instructions with minor modifications. Briefly, an additional dry spin at 21,000 ***g*** for 2 min was performed after washing DNA with Solution CB, and spin columns were incubated at 60 °C for 5 min to remove any residual ethanol. Purified DNA was eluted in 60 µl preheated Solution EB and incubated at room temperature for 5 min. Purified DNA was quantified using the Qubit dsDNA BR Kit (Fisher Scientific, UK) and Qubit machine (Fisher Scientific, UK), before being stored at −20 °C until required.

### Whole-genome sequencing, assembly and annotation

The genomes of 12 *Aeromonas* isolates were sequenced using the NovaSeq 6000 (Illumina) and MinION Mk1B (Oxford Nanopore Technology) platforms at the University of Exeter Sequencing Facility. The Nanopore reads were default filtered, and Illumina reads were adapter and quality trimmed using Fastp [24]. Trimmed reads were uploaded to the Bacterial and Viral Bioinformatics Resource Center (BV-BRC) version 3.35.5 [25], where they were used in hybrid assembly by Unicycler version 0.4.8 [26] with default settings. The genomic sequences were submitted to GenBank and annotated using the Prokaryotic Genome Annotation Pipeline [27]. Genome completeness and contamination were checked by checkM [28]. The genomic sequence and annotation information for isolates investigated in this study are publicly available via their accession numbers (Table 1).

### Species identification

The newly sequenced isolates were identified through the KmerFinder 3.2 database [29,31], hosted on the CGE platform (https://cge.food.dtu.dk/services/KmerFinder/). The assembled genomes in FASTA format were uploaded to the KmerFinder tool, and the ‘extended output’ was used to identify isolates, with the species assigned to the template with the highest score considered a positive match with the query genome.

### Core- and pan-genome analysis

The Roary pipeline [32] was employed to determine the core and pan-genomes (core plus accessory genome) of *A. dhakensis* and *A. hydrophila* isolates, using the GFF3 files generated by prokka 1.14.6 [33]. Phylogenetic trees were constructed from the output core genome alignment file using FastTree version 2.1.10 [34] on the Galaxy server (https://usegalaxy.org/), and pan-genomes were visualized using the roary_plots.py script, employing the absence and presence matrix of genes. A genome-wide association study was performed on the gene_presence_absence.csv file generated by Roary using Scoary [35]. A Fisher’s exact test and Benjamini–Hochberg false discovery rate adjustment were performed in Scoary to score each gene in the pan-genome against its potential correlation with either *Aeromonas* species.

### Genome-based phylogeny

*In silico* multi-locus sequence type (MLST) analysis was performed using assembled genomes in FASTA format, which were uploaded to the PubMLST database [36]. The ‘*Aeromonas*’ database was selected, which consists of alleles from six loci: *gltA, groL*, *gyrB*, *metG*, *ppsA* and *recA* [37]. Allele sequences and profile data were obtained by aligning loci using default settings on PubMLST. Single nucleotide polymorphisms (SNPs) were called against the core genome (generated with Roary) using snp-sites v2.5.1 [38]. The SNP-based alignment was used to infer a maximum likelihood (ML) phylogeny using RaxML v8.2.12 with the Gamma+GTR rate model [39]. The ML phylogeny was visualized in Figtree v1.4.4 and was mid-rooted.

### Accessory genome analysis

Genome assemblies were inspected for putative plasmids by screening contigs (<1 Mb) against a database of plasmid sequences from *A. dhakensis* or *A. hydrophila* recovered from Vietnam (six sequences). Contigs that returned no hits were then screened against the full plasmid database available on the NCBI (86,009 sequences). A similarity threshold of >97 % and an *E*-value of 0 was set to determine closely related plasmids. Plasmid contigs were visually checked for structure using the assembly graphs generated in BV-BRC and Bandage [40]. Further, contigs were screened for plasmid replicon genes using the PlasmidFinder database via ABRicate (https://github.com/tseemann/abricate) and applying a threshold of 60 % sequence coverage and 80 % sequence identity [41]. Features, including GC content and coding sequences (CDSs) of novel plasmids, were mapped using Proksee [42]. To investigate transmission of plasmids across the global *Aeromonas* genome collection, the ANI was calculated for each plasmid using FastANI (https://github.com/ParBLiSS/FastANI). Here, rows and columns represent each plasmid, and the ANI is calculated based on orthologous mapping of plasmid sequences. A heatmap of ANI distances was generated in RStudio v1.4.1717 using the ‘pheatmap’ package. Insertion sequences (ISs) were detected in assembled genomes using ISFinder (http://www-is.biotoul.fr) [43] and blastn, with an *E*-value threshold set to 1.00E-06 [17]. Genomic islands (GIs) were identified in assembled genomes and compared using IslandCompare [44]. Antimicrobial resistance genes (ARGs) were identified in chromosome and plasmid contigs by blast analysis against the Comprehensive Antibiotic Resistance Database [45] via ABRicate (https://github.com/tseemann/abricate). Virulence genes were identified by blast analysis against the Virulence Factor Database (VFDB) [46] via ABRicate. The presence of ARGs and virulence genes was determined based on a 60 % sequence coverage and 90 % sequence identity [47]. Protein secretion systems and related appendages were detected in genomes using MacSyFinder v2.1.4 [48] and annotated protein files. Systems were detected using default settings on ordered replicons and the model database TXSScan v 1.1.3.

### Statistical analysis

Statistical differences between the means of variables were determined by non-parametric Wilcoxon rank-sum or Kruskal–Wallis tests. A logistic principal component analysis (PCA) was conducted on a binary (0 and 1) matrix of genome components and accessory genes. A two-tailed Fisher’s exact test was performed to determine the statistical significance of distributions between categorical variables. All statistical analysis was performed on JMP Pro software v19.0.0, with significance determined when *P*<0.05.

## Results

### Genome characteristics

**Fig. 1.**
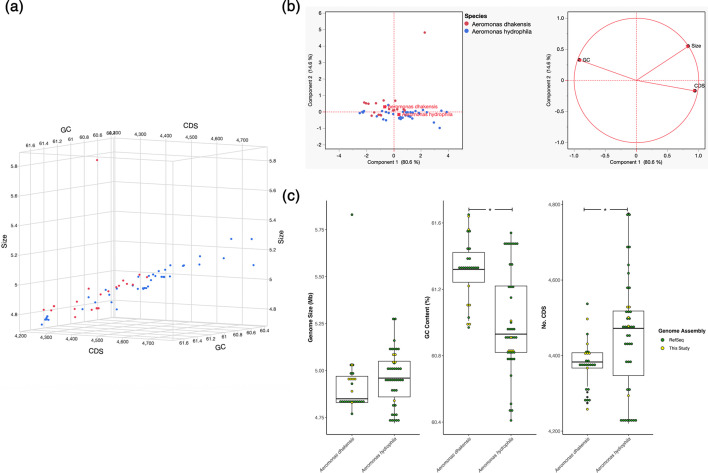
Genomic features of 26 *A. dhakensis* and 43 *A. hydrophila* isolates, recovered from various aquatic hosts and from different geographical locations. A three-dimensional plot of genomic features (genome size, GC content and CDS regions (a). Principal component analysis and canonical plot of genomic features in motile *Aeromonas* isolates (b). Comparison of genome size, GC content and CDS regions (c). In (a) and (b), dots coloured by species (red, *A. dhakensis*; blue, *A. hydrophila*). In Fig. 1c, dots were coloured based on genome assembly origin (green, RefSeq genome assemblies; yellow, genomes sequenced in this study). Significant differences (*P* < 0.05) in genomic features between species are denoted by *.

The general genomic features of 26 *A*. *dhakensis* and 43 *A*. *hydrophila* isolates from various aquatic hosts in different geographical locations are presented in Table 1. There was considerable overlap in genomic features between the two species investigated, with inter-individual variability also noted (Fig. 1a). Moreover, genome size was positively correlated with the number of CDS regions, whilst GC content was negatively correlated with both genome size and number of CDS regions (Fig. 1). The genomic features of isolates sequenced in this study fell within the expected range for the two species (Fig. 1). Mean genome size was not found to vary significantly between species (t-test, *P*>0.05), ranging from 4.93±0.03 and 4.96±0.03 Mb in *A. dhakensis* and *A. hydrophila*, respectively. Whilst mean GC content was found to be significantly higher in *A. dhakensis* (61.3±0.05 %) compared with *A. hydrophila* (61.00±0.04; t-test, *P*<0.0001), the total number of CDS regions was found to be significantly higher in *A. hydrophila* (4,448±19) genomes compared with those from *A. dhakensis* (4,379±25; t-test, *P*=0.03). All genomes sequenced in this study had a completeness of 100% and 0 % contamination, as revealed by CheckM.

### Core- and pan-genome analysis

**Fig. 2.**
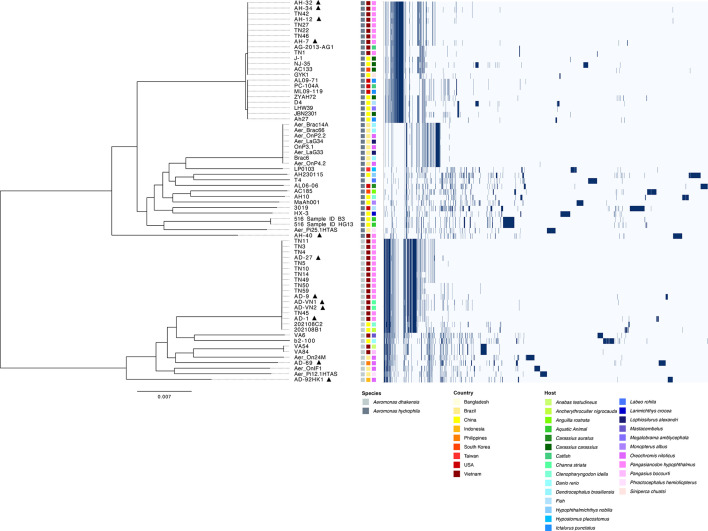
Pan-genome of *A. dhakensis* (*n*=26) and *A. hydrophila* (*n*=43) genomes from various aquatic sources. The figure shows the gene presence/absence matrix generated from the Roary pan-genome analysis pipeline. The left side illustrates the evolutionary relationship among isolates based on their core genomes. The matrix on the right side shows where genes were either present (dark blue) or absent. p denotes genomes sequenced in this study.

**Fig. 3.**
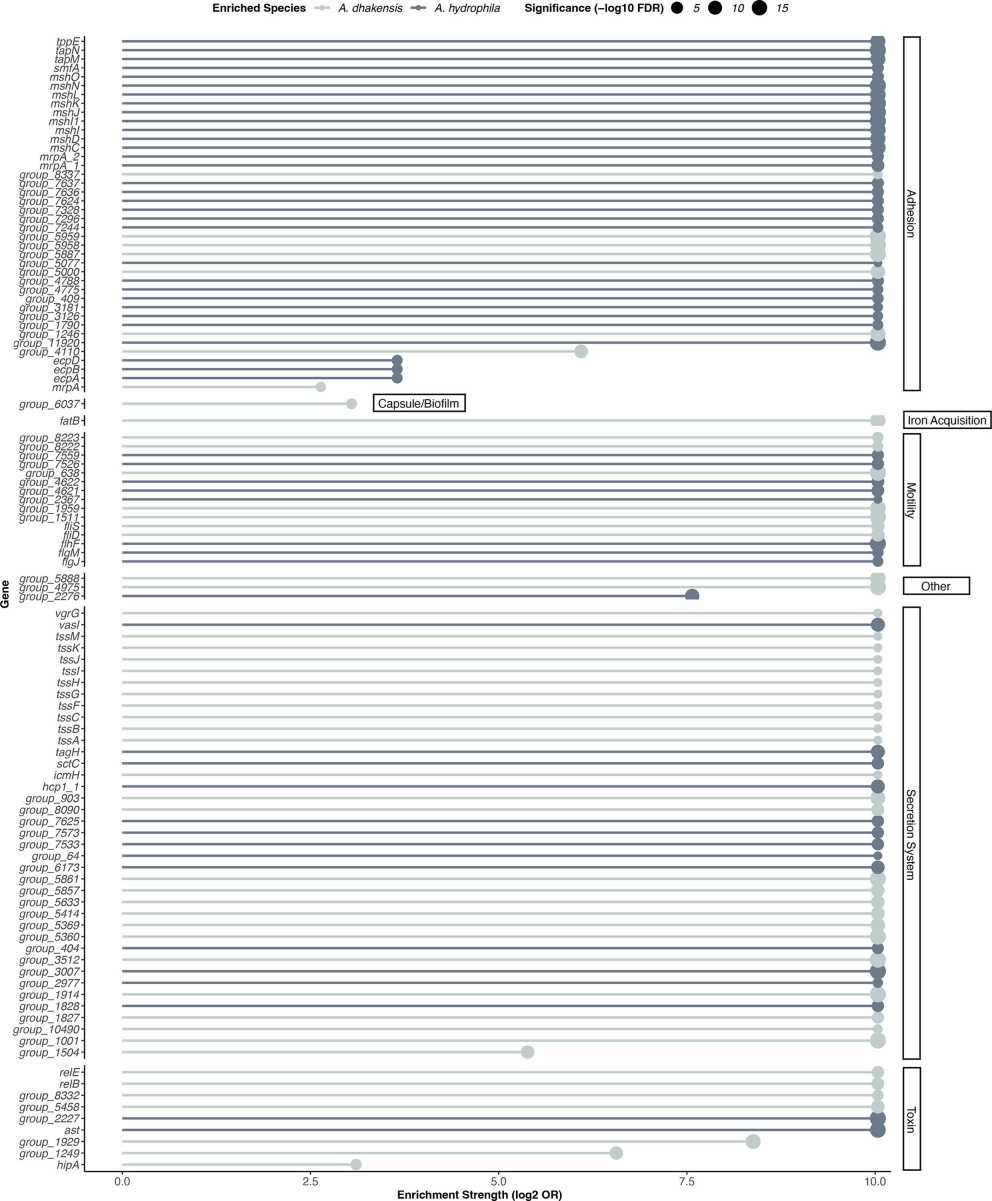
Gene-level enrichment of virulence factors between *A. dhakensis* and *A. hydrophila* from various aquatic sources. The lollipop plot shows virulence genes significantly associated with each species based on Scoary’s genome-wide association analysis. Stick length represents the magnitude of enrichment (based on the log_2_ odds ratio), point size corresponds to statistical significance (based on -log_10_ *P* values, adjusted using Benjamini–Hochberg method), and point colour indicates the species in which the gene was enriched. Genes are grouped by virulence category.

Pan-genome analysis revealed a total of 13,977 genes detected across the 69 *Aeromonas* genomes investigated (Fig. 2). Moreover, the core genome shared by both *A. dhakensis* and *A. hydrophila* represented 19 % (*n*=2,775 genes) of the total genes detected, whereas the cloud genome represented 58 % (*n*=8,038 genes) and was the largest component of the global gene pool amongst these two species. Accessory genomes showed distinct clustering based on species. Further, a genome-wide association study (GWAS) with Scoary identified a diverse repertoire of species-specific genomic markers associated with *A. dhakensis* (*n*=1,119 genes) and *A. hydrophila* (*n*=1,311 genes) (Table S1, available in the online Supplementary Material). Exploration of GWAS data revealed 108 genes to be virulence related and involved in adhesion (*n*=40), capsule/biofilm (*n*=1), iron acquisition (*n*=1), motility (*n*=15), secretion systems (*n*=39), toxins (*n*=9) and ‘other’ (*n*=3) (Fig. 3). Of these genes, *A. dhakensis* harboured a greater number of unique genes related to secretion systems (*n*=*26*) and toxins (*n*=7), compared with *A. hydrophila* (*n*=13 and 2, respectively). Further, unique *A. dhakensis* secretion system genes were primarily related to type VI secretion system (T6SS) (e.g. *tss*-family). In addition, the toxin–antitoxin (TA) system *RelBE* was also identified to be exclusive to *A. dhakensis*. In contrast, the thermostable cytotonic enterotoxin *ast* was found to be unique to *A. hydrophila. A. hydrophila* (*n*=32) also harboured a larger number of genes associated with adhesion compared with *A. dhakensis* (*n=*8). These genes were primarily related to pilus and fimbrial biogenesis, including *tapNM* and *msh-*family, respectively. Species-specific genes related to motility were evenly distributed between bacterial species, although pathways differed, with *fliSD* and *flhF* being exclusive to *A. dhakensis*, whereas the genes *flgM* and *flgJ* were unique to *A. hydrophila*.

**Fig. 4.**
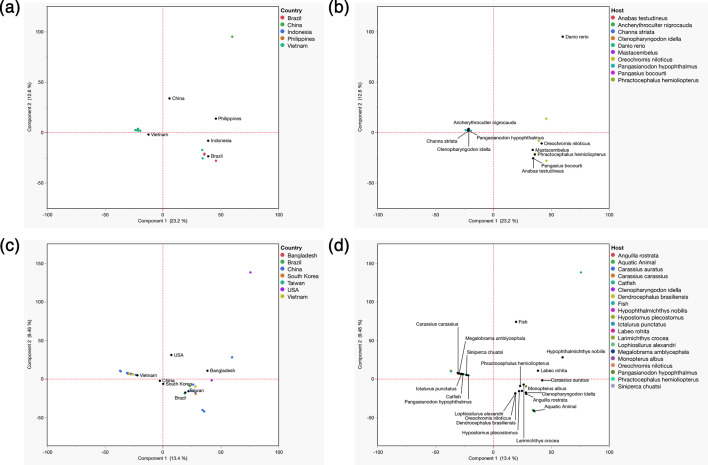
PCA of country and host influence on the accessory genome of *A. dhakensis* (*n*=26; a and b) and *A. hydrophila* (*n*=43; c and d) isolates recovered from various aquatic sources. The accessory genome of each isolate is coloured according to country (a and c) or host (b and d).

Further exploration of the pan-genomes at the species level found accessory genome composition of *A. dhakensis* to be more influenced by geographical location than host origin, confirmed by PCA (Fig. 4a and b). The majority of *A. dhakensis* isolates from China and Vietnam were found to share similar accessory genomes and clustered to the left of the PCA plot, whereas those recovered from Brazil clustered to the right. The *A. hydrophila* genomes showed more diversity in their accessory genomes (Fig. 4c and d), although isolates recovered from catfish species (e.g. *Ictalurus punctatus* and *Pangasianodon hypophthalmus*) had closely related accessory genomes (Fig. 4d).

### Genome-based phylogeny

**Fig. 5.**
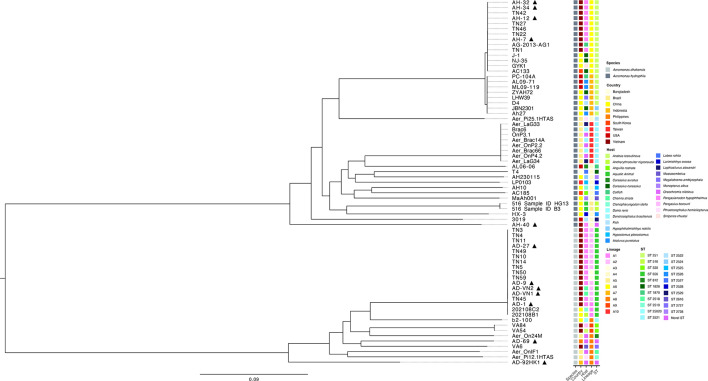
Population structure of 69 *Aeromonas* isolates recovered from various aquatic sources. ML phylogenetic tree of *A. dhakensis* (*n*=26) and *A. hydrophila* (*n*=43) genomes based on 403,996 high-quality single-nucleotide polymorphism positions called against the core genome. Species, country, host, lineage and ST for each isolate are provided at the same horizontal position. p denotes genomes sequenced in this study.

The 69 *Aeromonas* isolates were resolved into 21 sequence types (STs) and 3 novel STs, with a clear distinction between *A. dhakensis* and *A. hydrophila* (Fig. 5). Sequence type 656 dominated the *A. dhakensis* genome collection (65 %; *n*=17), where it was assigned to isolates from various host species in Vietnam (83 %; *n*=15) and China (67 %; *n*=2). In contrast, *A. dhakensis* isolates recovered from Brazil (ST 812, 2518, 2519), Indonesia (novel ST) and the Philippines (novel ST) were assigned to distinct, country-specific STs. Amongst the *A. hydrophila* genomes investigated, ST 251 dominated the collection (49 %; *n*=21) and was assigned to isolates recovered from China, South Korea, Vietnam and the USA. In contrast, isolates recovered from Bangladesh (ST 1826), Brazil (ST 2521, 2522) and Taiwan (ST 2528) were assigned to distinct STs that were country-specific, similar to findings from the *A. dhakensis* collection. On closer inspection, *A. hydrophila* isolate AH-40, sequenced in this study, was identified as a novel ST.

A total of 403,996 SNPs were detected across the core genome of the 69 *Aeromonas* isolates investigated. An ML tree based on the core genome SNPs is presented in Fig. 5. The core genome SNP phylogeny revealed a clear distinction between *A. dhakensis* and *A. hydrophila* isolates with five larger clades, consistent with *in silico* MLST results. *A. dhakensis* was dominated by a large clade, represented by isolates recovered from various host species in China and Vietnam, assigned to ST 656, and lineages A2 and A3. Three large clades were detected across *A. hydrophila* genomes. The largest clade was represented by isolates from various host species in Asia (China, Taiwan and Vietnam) and assigned to a single lineage (A6) and ST 251. The remaining ST 251 isolates were resolved into a closely related clade assigned to lineage A7, which also included the ST 2524 isolate JBN2301. This clade was found to be more globally distributed, represented by isolates from China as well as the USA. The third clade was revealed to be Brazil-specific, with isolates assigned to the lineage A10 and ST 2521.

### Mobilome

When all genomes were explored, 48 % (*n*=33) of isolates carried at least one plasmid type in their genome, whilst 39 % (*n*=27) of isolates carried at least two plasmid types. A significantly higher prevalence of isolates carrying at least one plasmid type was found for *A. dhakensis* (69 %; *n*=18) compared with *A. hydrophila* (35 %; *n*=15) (Fisher’s exact, two-tailed; *P*<0.01). Further, when geographical origin was explored, a significant association was detected between sampling country and plasmid-carrying isolates (Fisher’s exact, two-tailed; *P*<0.0001). Correspondence analysis showed a clear separation of Vietnam toward the plasmid-positive axis, where 90 % (*n*=26) of isolates from Vietnam carried at least one plasmid type. In contrast, all other countries clustered near the plasmid-negative axis, consistent with substantially lower plasmid prevalence (≤30 %).

**Fig. 6.**
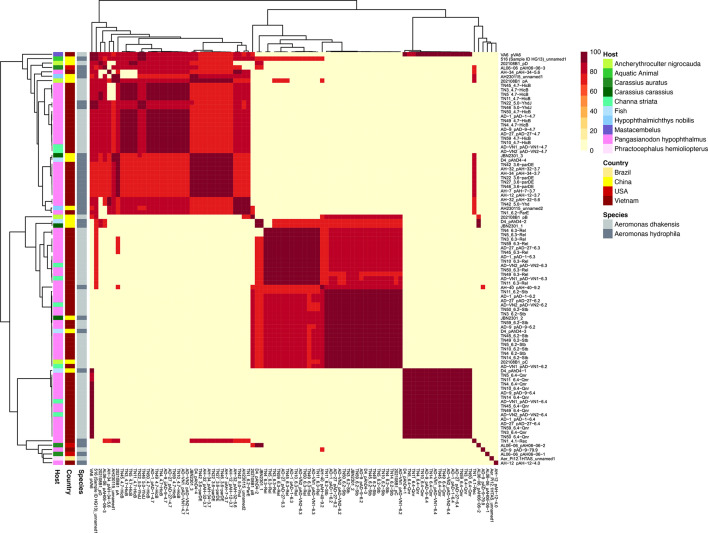
A dendrogram of average nucleotide identity for plasmid sequences (*n*=94) detected in *A. dhakensis* and *A. hydrophila* genomes. The heatmap shows a similarity matrix, which is coloured according to percentage nucleotide identity. On the left side, dendrogram nodes are coloured according to country, host and bacterial species origin for distinction (see legend).

**Fig. 7.**
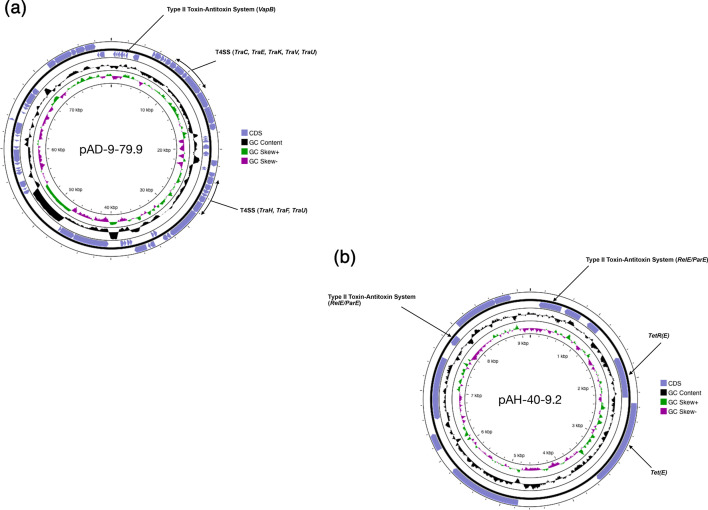
Map of novel plasmids pAD-9-79.9 (a) and pAH-40-9.2 (b) detected by whole-genome sequencing in this study. CDS regions, GC content and skew visualized using Proksee. Genomic features highlighted include type II TA systems, T4SS and tetracycline resistance gene *Tet(E)*.

The genomes of *A. dhakensis* sequenced in this study were found to carry between 0 and 4 plasmids (Table 2). No plasmids were detected in isolates AD-69 and AD-92HK1, recovered from the Philippines and Indonesia, respectively. Most *A. dhakensis* plasmids sequenced in this study were uniform in size at 4.7 Kb, 6.2 Kb, 6.3 Kb and 6.4 Kb and had high similarity (100 % sequence coverage and >99 % sequence identity) to the *A. dhakensis* plasmids 4.7-HicB, 6.2-Stb, 6.3-Rel and 6.4-Qnr. When compared with the global isolate collection, plasmids 4.7-HicB, 6.3-Rel and 6.4-Qnr were exclusive to *A. dhakensis* in Vietnam from both striped catfish (*Pangasianodon hypophthalmus*) and striped snakehead (*Channa striata*), where they shared >98 % ANI (Fig. 6). Whilst the plasmid 6.2-Stb was more frequently detected in *A. dhakensis* from Vietnam, a closely related (>98 % ANI) 6.2 Kb plasmid was also found in the genomes of two *A. hydrophila* isolates recovered from China. Isolate AD-9 harboured a novel, larger plasmid at 79.9 Kb, which comprised 78 CDS regions, including several genes from the type II TA system and a type IV secretion system (T4SS) (Fig. 7a). Between one and two plasmids were detected in the genomes of *A. hydrophila* sequenced in this study. Two plasmids were found to be identical (100 % sequence coverage and sequence identity) to other *A. hydrophila* plasmids (3.6-parDE and 5.0-YhdJ plasmids) previously detected and associated with striped catfish in Vietnam. Analysis by ANI revealed plasmid 3.6-parDE to be exclusive to *A. hydrophila* from striped catfish in Vietnam, where plasmids shared >98 % similarity, although a closely related (>91 % similarity) 6 Kb plasmid was also detected in *A. hydrophila* from non-catfish hosts in China. Isolate AH-12 harboured a 4.0 Kb plasmid, which was closely related (100 % sequence coverage and 99 % sequence identity) to a plasmid in *Edwardsiella ictaluri* from the USA and Vietnam. This was confirmed by ANI, which found the plasmid to be distinct (0 % similarity) from any other *Aeromonas* plasmid investigated. Further, a novel 9.2 Kb plasmid was detected in isolate AH-40, which encoded 12 CDS regions including several type II TA system and tetracycline resistance genes (Fig. 7b).

**Table 2.** blastn results of plasmids detected in *A. dhakensis* and *A. hydrophila* genomes sequenced in this study

Isolate	Species	Country	Host	Plasmid	Size (Kb)	Match	Species	Accession	*E*-value	Coverage	Match
AD-92HK1	*A. dhakensis*	Indonesia	*Oreochromis niloticus*	None detected	–	–	–	–	–	–	–
AD-69	*A. dhakensis*	The Philippines	*Oreochromis niloticus*	None detected	–	–	–	–	–	–	–
AD-VN1	*A. dhakensis*	Vietnam	*Channa striata*	pAD-VN1-6.4	6.4	6.4-Qnr	*A. dhakensis*	NZ_LR963083.1	0	100%	100%
				pAD-VN1-6.3	6.3	6.3-Rel	*A. dhakensis*	NZ_LR963080.1	0	100%	100%
				pAD-VN1-6.4	6.2	6.2-Stb	*A. dhakensis*	NZ_LR963081.1	0	100%	100%
				pAD-VN1-4.7	4.7	4.7-HicB	*A. dhakensis*	NZ_LR963082.1	0	100%	100%
AD-VN2	A*. dhakensis*	Vietnam	*Channa striata*	pAD-VN2-6.4	6.4	6.4-Qnr	*A. dhakensis*	NZ_LR963083.1	0	100%	100%
				pAD-VN2-6.3	6.3	6.3-Rel	*A. dhakensis*	NZ_LR963080.1	0	100%	100%
				pAD-VN2-6.2	6.2	6.2-Stb	*A. dhakensis*	NZ_LR963081.1	0	100%	100%
				pAD-VN2-4.7	4.7	4.7-HicB	*A. dhakensis*	NZ_LR963082.1	0	100%	100%
AD-27	*A. dhakensis*	Vietnam	*Pangasianodon hypophthalmus*	pAD-27-6.4	6.4	6.4-Qnr	*A. dhakensis*	NZ_LR963083.1	0	100%	100%
				pAD-27-6.3	6.3	6.3-Rel	*A. dhakensis*	NZ_LR963080.1	0	100%	99%
				pAD-27-6.2	6.2	6.2-Stb	*A. dhakensis*	NZ_LR963081.1	0	100%	100%
				pAD-27-4.7	4.7	4.7-HicB	*A. dhakensis*	NZ_LR963082.1	0	100%	100%
AD-9	A. dhakensis	Vietnam	*Pangasianodon hypophthalmus*	pAD-9-79.9	79.9	Novel	–	–	–	–	–
				pAD-9-6.4	6.4	6.4-Qnr	*A. dhakensis*	NZ_LR963083.1	0	100%	100%
				pAD-9-6.2	6.2	6.2-Stb	*A. dhakensis*	NZ_LR963081.1	0	100%	100%
				pAD-9-4.7	4.7	4.7-HicB	*A. dhakensis*	NZ_LR963082.1	0	100%	100%
AD-1	A. dhakensis	Vietnam	*Pangasianodon hypophthalmus*	pAD-1-6.4	6.4	6.4-Qnr	A. dhakensis	NZ_LR963083.1	0	100%	100%
				pAD-1-6.3	6.3	6.3-Rel	*A. dhakensis*	NZ_LR963080.1	0	100%	99%
				pAD-1-6.2	6.2	6.2-Stb	*A. dhakensis*	NZ_LR963081.1	0	100%	100%
				pAD-1-4.7	4.7	4.7-HicB	*A. dhakensis*	NZ_LR963082.1	0	100%	99%
AH-40	A. hydrophila	Vietnam	*Pangasianodon hypophthalmus*	pAH-40-9.2	9.2	Novel	–	–	–	–	–
AH-34	A. hydrophila	Vietnam	*Pangasianodon hypophthalmus*	pAH-34-5.6	5.6	5.0-YhdJ	*A. hydrophila*	NZ_LR963130.1	0	100%	100%
				pAH-34-3.7	3.7	3.6-parDE	A. hydrophila	NZ_LR963134.1	0	100%	100%
AH-32	A. hydrophila	Vietnam	*Pangasianodon hypophthalmus*	pAH-32-5.6	5.6	5.0-YhdJ	*A. hydrophila*	NZ_LR963130.1	0	100%	100%
				pAH-32-3.7	3.7	3.6-parDE	*A. hydrophila*	NZ_LR963134.1	0	100%	100%
AH-12	A. hydrophila	Vietnam	*Pangasianodon hypophthalmus*	pAH-12-4.0	4	19-pEI1	*Edwardsiella ictaluri*	NC_020281.1	0	100%	99%
				pAH-12-3.7	3.7	3.6-parDE	*A. hydrophila*	NZ_LR963134.1	0	100%	100%
AH-7	A. hydrophila	*Vietnam*	*Pangasianodon hypophthalmus*	pAH-7-3.7	3.7	3.6-parDE	*A. hydrophila*	NZ_LR963134.1	0	100%	100%

**Fig. 8.**
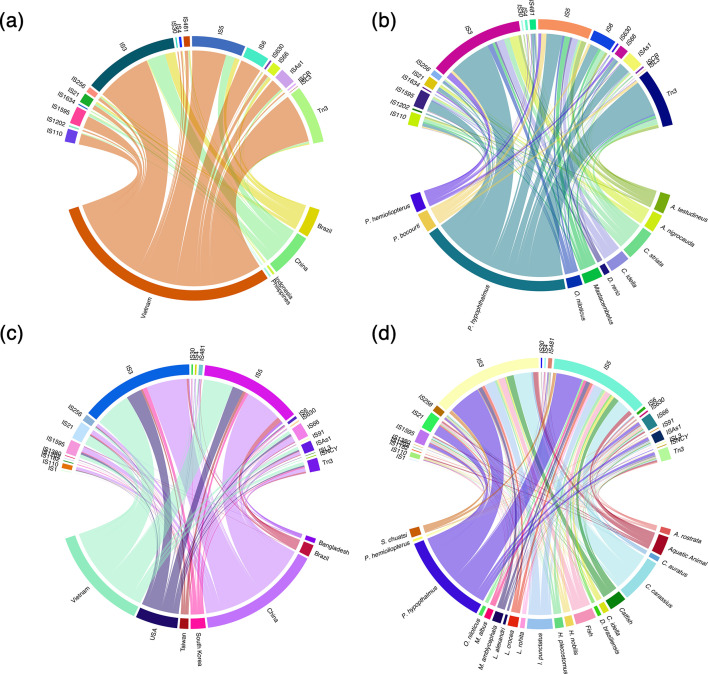
Chord diagram showing the distribution of IS families across the genomes of *A. dhakensis* (*n*=26; a and b) and *A. hydrophila* (*n*=43; c and d) recovered from various aquatic sources. For each species, chord diagrams illustrate the relationships between IS families and genome origin. Panels (a) and (c) show IS–country associations, and panels (b) and (d) show IS–host associations. Ribbon width is proportional to the number of genomes carrying a given IS family. Colours denote either country or host origin.

The number of ISs per genome in *A. dhakensis* (57±5 IS per genome) was significantly lower than in *A. hydrophila* (80±6 IS per genome) (Wilcoxon two-sample, *P*<0.001) (Table S2). When country-level association was further explored for *A. dhakensis*, isolates from China were found on average to carry a greater number of ISs (69±21 IS per genome), compared with isolates from other countries (8–63 IS per genome), although no significant difference was found (Kruskal–Wallis; *X*^2^=7.4, DF=4, *P*>0.05). The ISs detected in *A. dhakensis* genomes fell into 18 IS families, which were all detected in the chromosome. Further, seven of the IS families were also detected on various plasmids, including IS110, IS1202, IS3, IS481, IS5, IS66 and Tn3. The IS family Tn3 was conserved across all *A. dhakensis* genomes. Further, the families IS66, IS481 and TN3 were globally distributed and detected in at least one genome from each country investigated. Several IS families were also found to be country- or host-specific (Fig. 8a and b). The IS family ISL3 was only detected in genomes associated with China, whereas IS30, IS1202 and ISCR were exclusive to genomes from Vietnam, and IS1202 was exclusive to striped catfish-associated isolates only. Insertion sequences were detected in every *A. hydrophila* genome, with the number detected per genome ranging from 11 to 122 (Table S2). At the country level, a Kruskal–Wallis test showed significant differences in the average number of IS per genome between sampling countries (*X*^2^=27.15, DF=6, *P*=0.0001). Further, Dunn’s post hoc analysis revealed isolates from Vietnam (105±6 IS per genome) and China (98±3.7 IS per genome) to carry a significantly higher number of ISs average per genome compared with those from Brazil (14 IS per genome), whilst all other pairwise comparisons were not significant. A total of 21 IS families were detected across the *A. hydrophila* genomes. All families were detected in the chromosome of isolates; however, the families IS21 and Tn3 were also detected in the plasmids of some isolates. The IS families IS3, IS66 and Tn3 were conserved across *A. hydrophila* genomes. Three IS families (IS3, IS66 and Tn3) were globally distributed across all countries and associated with all host species investigated. Further exploration also revealed several IS families to be country- or host-specific (Fig. 8c, d). For example, IS1182 was exclusive to AC185, recovered from American eel (*Anguilla rostrata*) in South Korea, ISL3 was detected in isolate 3019 recovered from the USA and the family ISNCY was found only in the genomes of isolates recovered from striped catfish in Vietnam.

Exploration of GIs in the genome collection revealed *A. dhakensis* and *A. hydrophila* genomes to carry a similar number of GIs (15±1 and 17±1 GIs per genome, respectively) (Wilcoxon two-sample, *P*>0.05) (Tables S3 and S4). A Kruskal–Wallis test found significant differences between the number of GIs per genome and origin country, irrespective of bacterial species (*X*^2^=32, DF=8, *P*<0.0001). Comparatively, isolates from China (19±1 GIs per genome), USA (19±3 GIs per genome) and Vietnam (17±1 GIs per genome) carried a significantly higher number of GIs per genome compared with isolates from Brazil (11±1 GIs per genome) (Dunn’s test; *P*<0.002) average number of GIs per genome, irrespective of bacterial species. Comparative analysis of GIs in *A. dhakensis* and *A. hydrophila* genomes revealed isolates to carry various types of GIs, including virulence and resistance islands. Virulence islands encoding type I–IV and VI secretion systems, as well as type II and IV TA systems, *brkB*, flagellar, haemolysins, pili, V antigen, *VasX* and vertebrate lysozyme inhibitor, were frequently detected in isolate genomes, irrespective of bacterial species. However, country- and host-specific differences were also noted in the resistance and virulence islands carried by *A. dhakensis* or *A. hydrophila*. Isolates AD-69 and AD-92HK1 from the Philippines and Indonesia, respectively, carried 15 and 8 unique GIs, respectively, although these were predominantly metabolic islands and none encoded for resistance. In contrast, one GI (average size 21,057 bp) detected in 94 % (*n*=17) of *A. dhakensis* genomes from Vietnam encoded resistance to potentiated sulphonamides (*dfrA1* and *sul1*) and tetracyclines (*tet(A*)) (Fig. S1). Further, when host origin was explored, the isolates AD-VN1 and AD-VN2, recovered from striped snakehead, were found to carry a unique hybrid GI (average size 21,251 bp) encoding for DNA repair (*radC*) and the virulence factor *irmA*. In *A. hydrophila*, a resistance island detected in multiple genomes from China encoded genes against aminoglycosides (*AAC(6′)-Ib-cr5*), sulphonamides (*sul1*) and antiseptics (*qacE Delta 1*) (Fig. S2). Likewise, isolate AC185, recovered from South Korea, carried two islands encoding resistance to aminoglycosides (*aph(3′)-lb*), florfenicols (*floR*), potentiated sulphonamides (*dfrA12* and *sul1*) and tetracyclines (*tet(D*)) (Fig. S3). Isolate AH-40 carried a unique virulence island encoding a T6SS (e.g. *hcp1*, *tssB* and *vgrG*) not detected in any other isolate from Vietnam (Fig. S4). Further, a GI (average size 30,697 bp) detected in eight (73 %) *A*. *hydrophila* genomes from striped catfish in Vietnam encoded resistance to potentiated sulphonamides (*dfrA1* and *sul1*) and tetracyclines (*tet(A*)).

### Resistome

**Fig. 9.**
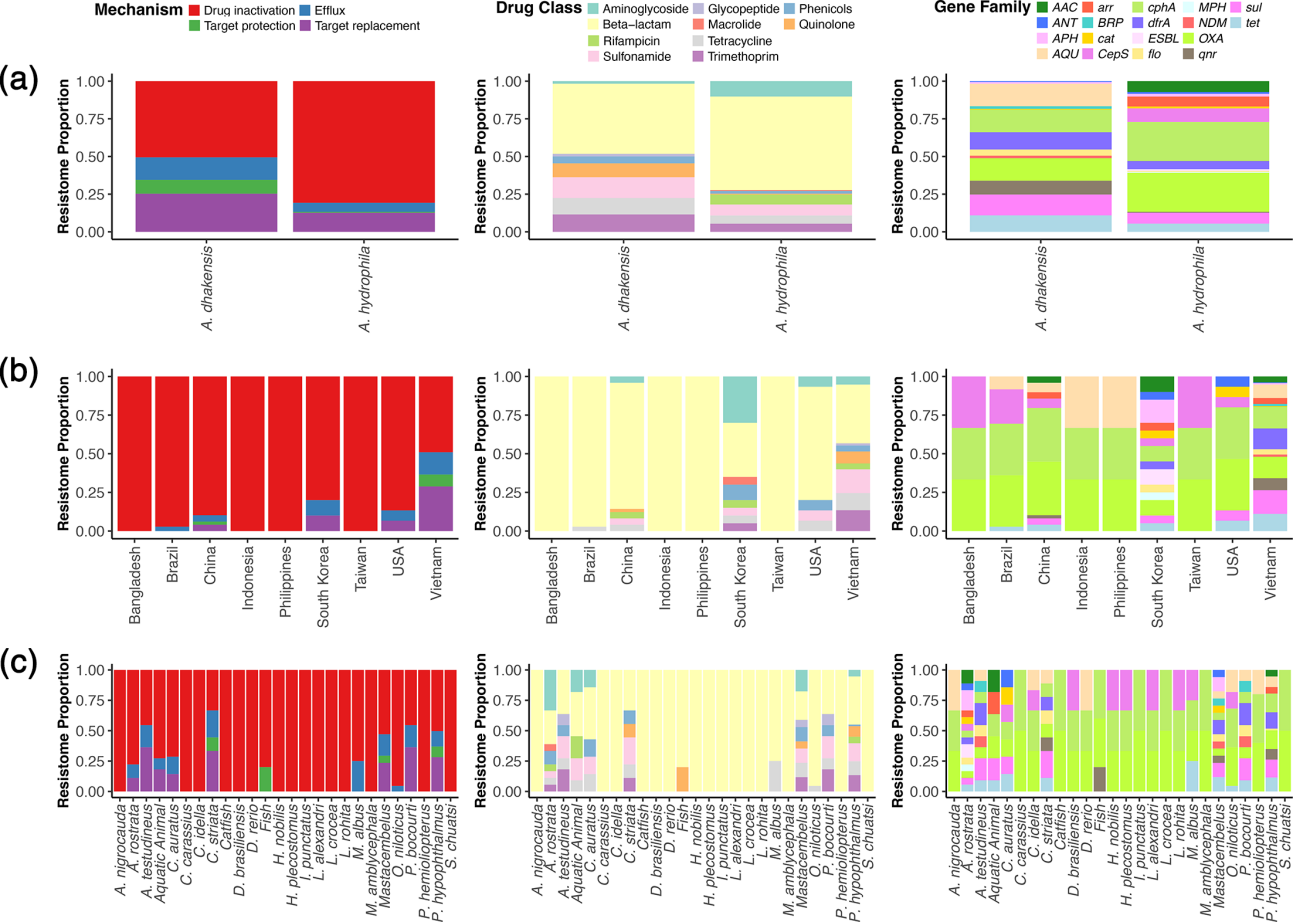
Resistome profiles of *A. dhakensis* (*n*=26) and *A. hydrophila* (*n*=43) genomes recovered from various aquatic sources. Plots illustrate the relative abundance of antimicrobial resistance genes across bacterial species (**a**), isolation country (**b**) and host origin (**c**). Stacked bars summarize the proportion of detected genes aggregated into resistance mechanisms, drug classes and gene families.

A total of 39 different ARGs were detected across the 69 isolates (Table S5). A mean of 5±3 ARGs was detected per genome, although *A. dhakensis* isolates (7±3 genes per genome) carried a significantly higher number of ARGs per genome compared with *A. hydrophila* (4±3 genes per genome) (Wilcoxon two-sample; *P*<0.0001). Whilst all four resistance mechanisms were found in both *Aeromonas* species, ARGs encoding for efflux, target protection and target replacement were more abundant in *A. dhakensis* genomes compared with *A. hydrophila* (Fig. 9a) (Fisher’s exact test, two-tailed; *P*<0.0001). Although *A. hydrophila* displayed a higher relative proportion of drug-inactivation ARGs, Fisher’s exact test showed no significant difference in genome-level prevalence between both species (*P*>0.05). When ARGs were aggregated by drug class, ARGs conferring resistance to phenicol, quinolone, sulphonamide, tetracycline and trimethoprim drug classes were more abundant in *A. dhakensis* compared with *A. hydrophila* (Fisher’s exact test, two-tailed; *P*<0.05). In contrast*,* ARGs encoding resistance to aminoglycosides were more frequently detected in *A. hydrophila* (Fisher’s exact test, two-tailed; *P*<0.001). Further, resistance to glycopeptide and rifamycin was exclusive to *A. dhakensis* and *A. hydrophila*, respectively. The beta-lactamase gene *OXA-724* was conserved across all genomes, irrespective of bacterial species. However, other beta-lactamase genes were identified to be species-specific. Specifically, *A. dhakensis* carried the families *AQU* and *NDM*, whereas *A. hydrophila* carried *CepS* (Fisher’s exact test, two-tailed; *P*<0.05). Other species-specific gene families included *AAC* (*A. hydrophila*; aminoglycoside), *BRP* (*A. dhakensis*; glycopeptide) and *arr* (*A. hydrophila*; rifamycins). Whilst not unique*,* the gene families *dfrA*, *qnr*, *sul* and *tet*, encoding resistance to trimethoprim, quinolones, sulphonamides and tetracyclines, respectively, were more abundant in *A. dhakensis* genomes compared with those belonging to *A. hydrophila* (Fisher’s exact test, two-tailed; *P*<0.001).

Since different ARGs might be associated with genome origin, we also explored the influence of country and host on ARG presence and resistome profiles. When country-level data were explored, a Kruskal–Wallis test found a significant difference between the number of ARGs per genome and sampling country for *A. dhakensis* only (*X*^2^=17.22, DF=4, *P*<0.01), whilst no significant difference was observed for *A. hydrophila* (*X*^2^=10.71, DF=6, *P>*0.05). When ARGs were aggregated by resistance mechanism, the genomes from China and Vietnam showed the greatest diversity with all four resistance mechanisms detected, followed by South Korea (*n*=3) and USA (*n*=3) (Fig. 9b). Further, all four resistance mechanisms were detected at significantly higher frequencies in Vietnam compared with other countries (Fisher’s exact test, two-tailed; *P*<0.001). When ARGs were aggregated by drug class, ARGs encoding resistance to beta-lactam antibiotics were the most abundant, irrespective of sampling country. However, country-specific differences were also noted for several drug classes, including resistance to aminoglycosides, which was significantly associated with South Korean genomes and associated with the *APH* gene family (Fisher's exact test; *P*<0.05). Likewise, a significantly higher proportion of genomes from Vietnam carried the gene families *dfrA*, *qnr*, *sul* and *tet*, which confer resistance to quinolone, sulphonamide, tetracycline and trimethoprim classes (Fisher’s exact test, two-tailed; *P*<0.01). Several gene families were country-specific, including *BRP* (glycopeptide) and *NMD* (beta-lactam), which were exclusive to genomes from Vietnam. Likewise, ESBL (beta-lactams) and *MPH* (macrolide) ARGs were only found in genomes from South Korea. The *arr* family (rifampicin) was exclusive to Asia, where it was detected in genomes from China, South Korea and Vietnam, but absent in those from Brazil and the USA.

At the host level, drug inactivation was the most prevalent resistance mechanism detected across genomes (Fig. 9c). However, all four resistance mechanisms were detected in the bacterial genomes associated with three host species: *Mastacembelus,* striped catfish and striped snakehead. When ARGs were aggregated by drug class, ARGs encoding resistance to beta-lactam antibiotics were the most abundant irrespective of host origin. Further, the genomes from 58 % (*n*=15) of host species carried ARGs encoding resistance to beta-lactam antibiotics only. Macrolide resistance was exclusive to the genome of AC185 from the American eel, encoded by *MPH* genes. Further, when ARGs were aggregated by gene family, the genome of AC185 also displayed the greatest resistome diversity with ARGs assigned to 14 families. This was followed by *Mastacembelus* (*n*=13) and striped catfish (*n*=10).

### Virulome

**Fig. 10.**
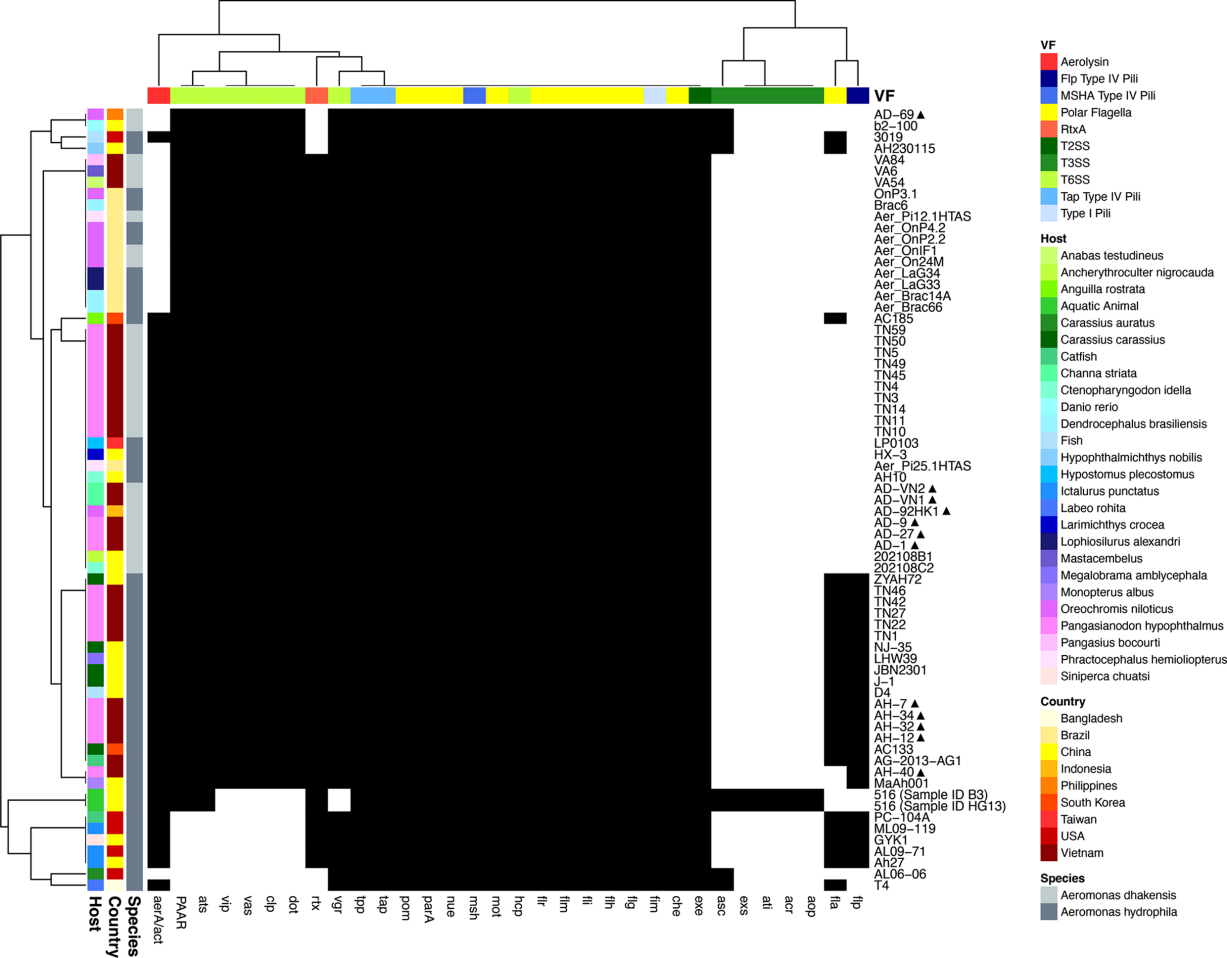
A heatmap illustrating the distribution of virulence gene families across *A. dhakensis* (*n*=26) and *A. hydrophila* (*n*=43) genomes from various aquatic sources. Rows represent individual genomes and columns show the presence (black) or absence (white) of 32 gene families. Row annotations correspond to genome origin (bacterial species, country and host). Column annotations indicate virulence factor (VF) classification and are coloured based on general virulence category, including adherence (blue), effector delivery system (green), exotoxin (red) and motility (yellow). p denotes genomes sequenced in this study.

A total of 194 different virulence genes were detected across the global *Aeromonas* genome collection (Table S6), which comprised adherence (*n=*54; 28%), effector delivery systems (*n=*77; 40%), exotoxins (*n=*7; 4%) and motility (*n=*56; 29%) associated genes. The number of genes per genome ranged from 103 to 158, although on average, *A. hydrophila* genomes carried a significantly higher number of genes (*n*=142±14 genes) compared with *A. dhakensis* (*n*=111±2 genes) (Wilcoxon two-sample test; *P*<0.0001). Virulence genes were grouped into 32 gene families, and the distribution of these families across genomes is presented in Fig. 10. Sixteen gene families were conserved across the genome collection, irrespective of bacterial species. The major virulence factors identified in all genomes were associated with MSHA type IV pili (e.g. *msh*), polar flagella (e.g. *che*, *flg*, *flh*, *fli*, *flm*, *flr*, *mot*, *nue*, *parA* and *pom*), Tap type IV pili (e.g. *tpp* and *tap*), type I pili (e.g. *fim*), type II secretion system (T2SS, e.g. *exe*) and T6SS (e.g. *hcp*). Whilst no genes were unique to *A. dhakensis* genomes, the gene families *fla* and *flp* associated with polar flagella and MSHA type IV pili, respectively, were exclusive to *A. hydrophila* genomes (Fisher’s exact test, two-tailed; *P*<0.0001).

As shown in Fig. 10, the genomes sequenced in this study tended to cluster together with genomes from similar geographic or host origins, broadly displaying similar virulence profiles. However, whilst origin-associated similarities in the virulome of isolates were evident, the profiles often overlapped across distinct groups. Notably, one of the newly sequenced genomes, AH-40, deviated from the general trend observed for *A. hydrophila* in Vietnam, supporting trends observed in phylogeny previously (Fig. 5). Instead, AH-40 displayed a similar virulence profile to isolate MaAh001, recovered from Asian swamp eel (*Monopterus albus*) in China. Specifically, both AH-40 and MaAh001 differed from other *A. hydrophila* from similar regions by lacking *fla* genes. When genome origin was further explored, several genes were found to be country and lineage-specific. Indeed, the *flp* gene associated with type IV pili was significantly associated with ST 251 (Fisher’s exact test, two-tailed; *P*<0.0001), specifically genomes from China, South Korea, USA and Vietnam (Fisher’s exact test, two-tailed; *P*<0.05). Likewise, significant lineage and country associations (Fisher’s exact test, two-tailed; *P*<0.0001) were also observed in the prevalence of the exotoxin *aerA/act*, where it was conserved in ST 251 and ST 656, whereas the prevalence of *aerA/act* was significantly lower in Brazil compared with other countries. At the host level, *aerA/act* was conserved in isolates recovered from channel catfish (*Ictalurus punctatus*), crucian carp (*Carassius carassius*), striped catfish and striped snakehead, but had significantly lower prevalence in genomes associated with fairy shrimp (*Dendrocephalus brasiliensis*) and Nile tilapia (*Oreochromis niloticus*) (Fisher’s exact test, two-tailed; *P*<0.0001). Genes associated with a second exotoxin, *rtxA*, were also detected in 90 % (*n*=67) of genomes; however, differences were noted in operon completeness (Table S6). Specifically, a complete exotoxin *rtxA* operon (*rtxA/B/C/D/E/H*) was significantly associated with Brazil, where it was conserved in ST 2521 (Fisher’s exact test, two-tailed; *P*<0.0001). A complete *rtxA* operon was also detected in other countries, albeit significantly lower in prevalence (Fisher’s exact test, two-tailed; *P*<0.0001). This included Vietnam, where it was detected in three *A. dhakensis* isolates (VA6, VA54, VA84), as well as several *A. hydrophila* isolates in China (21 %; *n*=3) and South Korea (50 %; *n*=1) carried the full *rtx* operon. Finally, several type III secretion system (T3SS) genes (e.g. *acr*, *aop*, *ati* and *exs*) were exclusive to two genomes recovered from China.

### Macromolecular systems

**Fig. 11.**
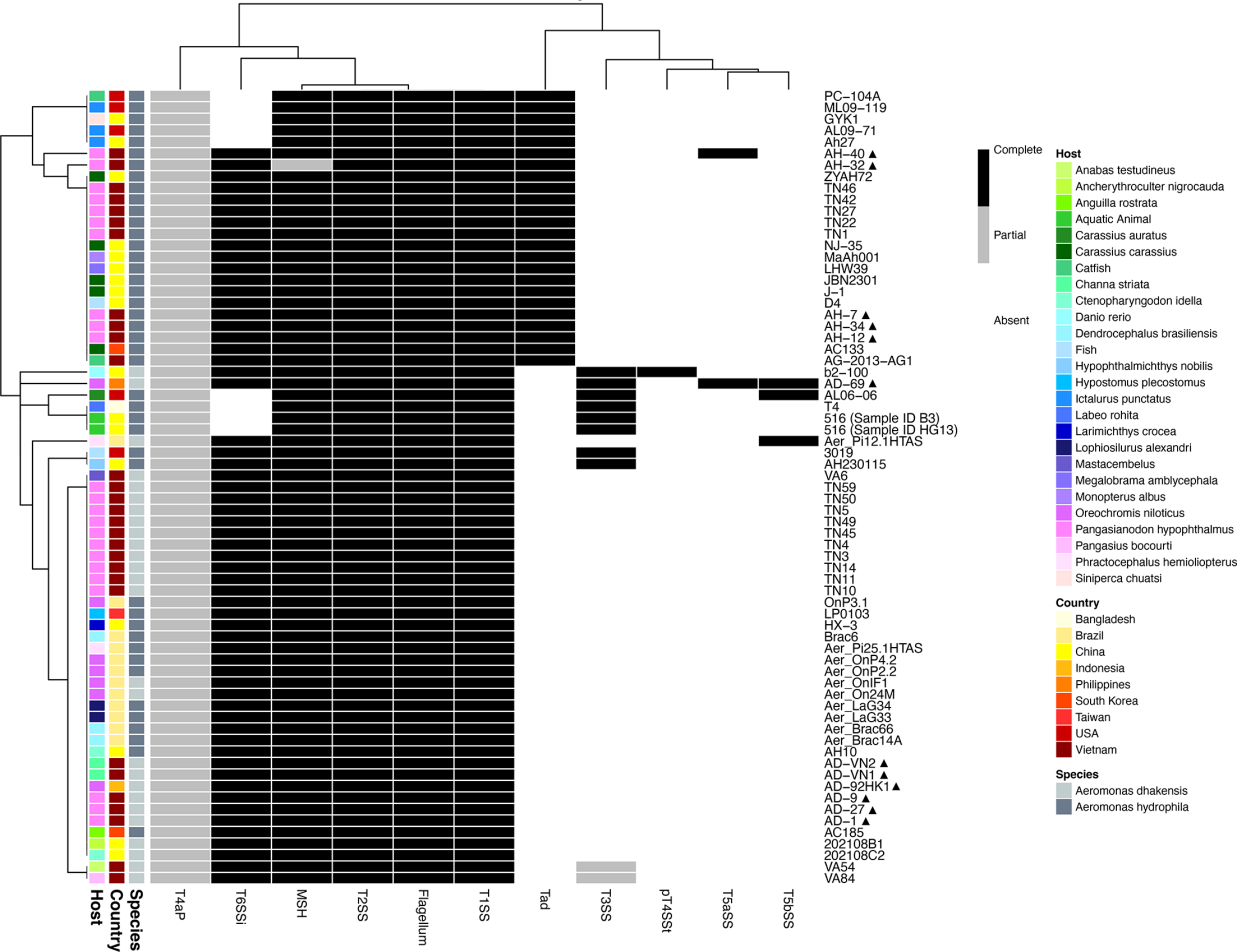
A heatmap illustrating the distribution of macromolecular systems in *A. dhakensis* (*n*=26) and *A. hydrophila* (*n*=43) genomes from various aquatic sources. Rows represent individual genomes, and columns show the presence or absence of 11 systems. Row annotations correspond to genome origin (bacterial species, country and host). Cells are coloured based on system completeness: complete (required number of mandatory genes detected: black), partial (≥ 1 but less than min. required mandatory genes detected: grey) and absent (no mandatory gene detected: white). Systems (*n*=min. required mandatory genes) include flagellum (*n*=11), MSH pilus (*n*=5), type I secretion system (T1SS; *n*=3), type II secretion system (T2SS; *n*=4), type III secretion system (T3SS; *n*=9), type IVa pilus (T4aP; *n*=6), type IV secretion system (pT4SSt; *n*=1), type Va secretion system (T5aSS; *n*=1), type Vb secretion system (T5bSS; *n*=1), type VI secretion system (T6SSi; *n*=14) and tight adherence (Tad) pilus (*n*= 7). p denotes genomes sequenced in this study.

A total of 11 macromolecular systems were detected across the genome collection, albeit at varied levels of completeness (Fig. 11, Table S7). Three complete systems were conserved across genomes, including the flagellum, the type I secretion system and the type II secretion system. A complete mannose-sensitive haemagglutinin (MSH) pilus system was also detected in 99 % (*n*=68) of genomes investigated. However, only a partial MSH system was detected in the genome of AH-32, sequenced in this study. Whilst VFDB screening detected all mandatory *msh* genes, including *mshA* in AH-32 (Table S7), MacSysFinder failed to recognize *mshA* and thus a complete MSH system in this isolate. All genomes carried core elements of type IVa pilus (T4aP), although they all lacked the mandatory gene *pilA* and thus only a partial T4aP system was conserved. A complete T3SS and T6SS were detected in 12 % (*n*=8) and 87 % (*n*=60) of genomes investigated. Further, Fisher’s exact tests found the prevalence of these systems to be significantly associated with country (two-tailed; *P*<0.01). Specifically, complete T3SS were more frequently detected in genomes recovered from Bangladesh, China, Indonesia and the USA. In contrast, the prevalence of complete T6SS was significantly lower in Bangladesh and the USA. The Tad (tight adherence) pilus was exclusive to *A. hydrophila*, where it was detected in 56 % (*n*=24) of genomes. Further, Tad showed significant geographic (Fisher’s exact test, two-tailed; *P*<0.05) and lineage (Fisher’s exact test, two-tailed; *P*<0.0001) associations, being significantly prevalent in China, South Korea, the USA and Vietnam, as well as being conserved across all isolates of ST 251. Complete pT5SSt and type V secretion system (T5SS) were rare, detected in 2 % (*n*=1), 3 % (*n*=2) and 4 % (*n*=3) of isolates, respectively. Whilst pT4SSt was exclusive to China, the T5aSS and T5bSS systems were shared across countries and hosts. The genome of AD-69, sequenced in this study, was unique in carrying complete sets of both T5SS.

## Discussion

Analysis of the pan-genome in global *Aeromonas* isolates revealed large genomic variability with only a small set of core genes (19%) shared between all *Aeromonas* genomes investigated (Fig. 2). These findings agree with those reported previously for *Aeromonas* [49]*.* Whilst we recognize the cited study was based on a broader multi-species dataset, the large genomic variability observed in both studies highlights the ability of this genus to adapt and occupy diverse ecological niches, with our analysis specifically reflecting *A. dhakensis* and *A. hydrophila*. Indeed, *Aeromonas* can be found in various aquatic environments, as well as food (fruits, vegetables, dairy and meat) and terrestrial animals [1]. In the work presented, the presence of closely related pan-genomes in isolates from similar geographical regions and fish species (Figs2  4) suggests a divergent evolution and local adaptation of these two species, with pan-genomes shaped more by the immediate environment. Environmental niche and local geography have been shown to shape the pan-genomes of other bacterial pathogens, including *E. ictaluri* [23], *Escherichia coli* [50] and *Salmonella enterica* [51]*,* and highlight the importance of local disease management strategies in controlling the spread of MAS.

Genomic comparisons of circulating *Aeromonas* populations revealed highly divergent clusters, dominated by ST 656 and ST 251 for *A. dhakensis* and *A. hydrophila*, respectively (Fig. 5). The ST 656 dominates the *A. dhakensis* recovered from the striped catfish sector in Vietnam [52]; however, findings from this study suggest that ST 656 has a wider regional distribution within Asia, with this ST also detected in China. This study also detected novel STs in the newly sequenced genomes of the *A. dhakensis* isolates AD-92HK1 and AD-69 from Indonesia and the Philippines, which were distinct from the STs of other isolates recovered from Nile tilapia (*Oreochromis niloticus*). Indeed, all four *A. dhakensis* genomes associated with Nile tilapia differed in every allele examined, highlighting a high level of genetic divergence within *A. dhakensis*, irrespective of host, and would suggest that AD-92HK1 and AD-69 may have undergone adaptation to specific environmental conditions within their respective countries; however, additional strains would be beneficial to confirm. The presence of ST 251 in China, South Korea, the USA and Vietnam suggests a global distribution of clonal *A. hydrophila* populations. Previous reports from Vietnam revealed the ST 251 to dominate *A. hydrophila* populations causing disease in striped catfish farms [15,52]. Whilst our findings would agree in principle, in our study, isolated AH-40 was confirmed as belonging to a novel ST. Further analysis of the allelic profile of AH-40 revealed this isolate to be distinct from all other isolates recovered from striped catfish in Vietnam, differing in every allele examined. A study by [53] also identified novel STs in isolates recovered from diseased catfish in Vietnam, although these isolates differed from AH-40 in the alleles of *gyrB, groL*, *metG* and *ppsA*. These findings would suggest that *A. hydrophila* populations circulating the Vietnamese striped catfish sector are more divergent than previously assumed and warrant increased microbial surveillance studies to better understand the prevalence of these novel STs associated with disease.

The influence of local geography in shaping the population dynamics of *Aeromonas* isolates was also evident in the different mobilome elements found across genomes. Indeed, ANI revealed several country- or host-specific plasmids associated with numerous genomes (Fig. 6). Likewise, statistical analysis revealed that isolates from Vietnam were significantly more likely to harbour plasmids, compared with genomes from other countries, such as Indonesia and the Philippines, where genomes lacked any plasmids. The number and identity of plasmids detected in *A. dhakensis* and *A. hydrophila* from Vietnam agree with previous reports [52]. However, the presence of a novel plasmid in isolate AD-9 (Fig. 7a) and an *E. ictaluri* plasmid in isolate AH-12 (Table 2) also reflects unexpected diversity in the plasmid reservoir of circulating *Aeromonas* populations within Vietnam. Together, these findings would suggest the potential for large-scale horizontal gene transfer within these systems, which could drive future changes in AMR and pathogenicity.

A closer inspection of the resistome of *A. dhakensis* and *A. hydrophila* revealed a high prevalence of antibiotic inactivation genes in these genomes*,* supporting our existing understanding of common AMR mechanisms in Gram-negative bacteria [54]. The gene *OXA-724*, which encodes a class D *β*-lactamase, was conserved across all genomes investigated (Table S5), in agreement with previous reports [52,55]. However, further analysis showed country-specific resistome profiles with varying degrees of predicted resistance levels. Specifically, the newly sequenced *A. dhakensis* isolates from Indonesia and the Philippines, as well as *A. hydrophila* from Bangladesh and Taiwan, carried three ARGs in their genomes, encoding resistance to beta-lactam compounds only (Fig. 9b). These findings also support the phylogenetic clustering and distinct ST assignment found for these isolates (Fig. 5), indicating that AMR drivers are likely clade-associated. In contrast, isolates recovered from other regions in Asia demonstrated the potential for multidrug resistance (MDR), carrying genes encoding resistance to at least three antibiotic classes [56]. In Vietnam, most isolates (86 %; *n*=25) carried ARGs against more than five antibiotic classes, including aminoglycoside, fluoroquinolone, potentiated sulphonamides (inc. diaminopyrimidine) and tetracycline (Fig. 9b). Likewise, several genomes associated with China (12 %; *n*=2) and South Korea (50 %; *n*=1) also presented MDR, encoding ARGs against aminoglycosides, potentiated sulphonamides and tetracyclines (Fig. 9b). These findings support existing reports across Asia of MDR development in bacterial pathogen populations associated with aquaculture, where these antibiotic classes are frequently used [15,57,60]. This contrasted with trends observed in Brazil and the USA, where low or no incidence of MDR was observed in isolates recovered from these countries (Fig. 9b). We hypothesize that these differences in MDR detected across regions reflect differences in antimicrobial stewardship and disease management practices. Whilst these will undoubtedly play a role, the factors and sources influencing AMR leading to the emergence of MDR isolates within aquatic systems, including fish farms, are complex. Surveillance and open data on antimicrobial usage are crucial to disentangle this interplay and better understand AMR drivers across the global aquaculture sector.

*A. dhakensis* and *A. hydrophila* carry an extremely diverse repertoire of genes within their virulome [61]. In this study, effector delivery systems were found to dominate the virulome of *Aeromonas* species investigated (Fig. 10). As these systems allow for efficient and targeted manipulation of the host [62], dominance of this pathway in *Aeromonas* from aquatic environments would be particularly advantageous where hosts may be more diverse or readily accessible to the bacterium. In addition, *A. hydrophila* was found on average to carry a significantly higher number of virulence genes within its genome compared with *A. dhakensis*. Further analysis also revealed *fla* and *flp*-related virulence factors to be exclusive to *A. hydrophila*. Whilst little is known about the role of *flp* pili in the virulence of motile *Aeromonas* [63], the flp pili facilitate host invasion and defence in other aquatic bacterial pathogens [64]. Likewise, the lateral flagella encoded by fla genes serve important functions for *Aeromonas* in adhesion, motility and biofilm formation [65]. In addition, findings from GWAS of the *Aeromonas* pan-genome in this study also identified the heat-stable cytotonic enterotoxin ast, which is involved in the pathophysiology of MAS clinical signs [57], to be specific to *A. hydrophila*. Together, these findings would, therefore, suggest a greater virulence potential for *A. hydrophila* compared with *A. dhakensis*, contradicting anecdotal evidence of *A. dhakensis* being more prevalent in MAS outbreaks in different aquatic hosts [15,52, 53, 66, 67]. However, these findings do support other studies reporting a higher prevalence of *A. hydrophila* associated with MAS in other fish species [68,69]. In addition, findings also demonstrated that the *Aeromonas* virulome was shaped by host and geographical pressures, echoing the phylogenetic topology. For example, whilst ST 2521 isolates from Brazil lacked the exotoxin aerA/act, they all carried a complete rtx operon in their genome (Table S7). In contrast, whilst aerA/act was conserved in dominant STs (ST 251 and ST 656) circulating in China, the USA and Vietnam (Fig. 10), the presence of a complete *rtx* operon was rare in these countries. Whilst both systems contribute to the clinical signs observed in MAS [61], these genes have distinct functions in the bacterium’s pathogenesis. Together, the findings from this study, therefore, suggest potential clade-specific virulence repertoires and reflect the complex interplay between environment, host susceptibility and pathogen virulence. Given the diverse host range of both *A. dhakensis* and *A. hydrophila*, the virulome will undoubtedly be influenced by differences in host defence mechanisms and farming conditions. Surveillance and challenge studies are, therefore, required to better understand these interactions and identify drivers behind the divergence in the virulome*.*

Macromolecular secretion systems are vital to the normal functioning of the bacterial cell, as well as promoting pathogenesis in bacterial pathogens through the transport of small molecules, nucleic acids and proteins, e.g. toxins [62,70]. In this study, we detected gene clusters associated with a variety of systems, including flagella, Tad pili and type secretion systems (I–VI) (Fig. 11). Whilst flagella genes were conserved across all *Aeromonas* genomes investigated, genes associated with Tad pili were exclusive to *A. hydrophila* genomes and conserved in ST 251, suggesting that the presence of this system is largely lineage-dependent. The Tad pili are an Archaea-derived pili, acquired through horizontal gene transfer, and facilitate a variety of processes, including motility, adherence and DNA uptake in the host bacterium [71]. In *A. hydrophila*, Tad has been shown to function in biofilm formation and virulence in channel catfish [72], thus highlighting the importance of this gene cluster in the pathogenicity of aquatic isolates affecting different farmed fish species. Local geography was also found to shape macromolecular systems in *Aeromonas* genomes, as the presence of a complete T3SS was isolated to a few isolates (12 %; *n*=8) across Bangladesh, China, Indonesia and the USA, yet all isolates from Vietnam, irrespective of bacterial species, lacked this machinery. In other bacteria, the T3SS assembles into a needle-like structure on the surface of the bacterial cell and allows the transfer of effector proteins into the target cell and allows host cell colonization [73]. The absence of such a vital secretion system in some genomes would, therefore, suggest a reliance on alternative virulence pathways in these isolates. Likewise, isolate AD-69 was unique in carrying both T5aSS and T5bSS (Fig. 11). The low prevalence of T5SS across the genomes investigated would, therefore, suggest this secretion system is of limited importance in the virulence of *A. dhakensis* or *A. hydrophila*, supporting previous reports in other *Aeromonas* species [19].

In conclusion, comparative genomic analysis in this study has contributed to a better understanding of *A. dhakensis* and *A. hydrophila* populations circulating in the global aquatic environment. Findings from this study suggest that whilst *A. dhakensis* comprises distinct clades, *A. hydrophila* is largely dominated by a single clade that is found across multiple continents and host species. However, local geography and host species exert significant influence on the genotypes of both *A. dhakensis* and *A. hydrophila*, likely driven by multiple factors, including anthropogenic pressure, environmental conditions within production systems and host genotypes. As evident by the high prevalence of MDR genomes in certain countries and niche-specific virulome profiles observed in this study, these pressures will likely facilitate divergence in both AMR and pathogenesis of *Aeromonas* populations that could lead to increases in disease burden or treatment failure within production systems. Our results, therefore, provide a critical framework to support future surveillance and comparative genomics studies, to better inform targeted and effective control strategies for MAS in aquaculture.

## Supplementary material

10.1099/mgen.0.001618 Uncited Supplementary Material 1.

## Abbreviations

AMR: antimicrobial resistance
ANI: average nucleotide identity
ARGs: antimicrobial resistance genes
BV-BRC: bacterial and viral bioinformatics resource center
CDSs: coding sequences
GIs: genomic islands
GWAS: genome-wide association study
ISs: insertion sequences
MAS: motile *Aeromonas* septicaemia
MDR: multidrug resistance
ML: maximum likelihood
MLST: multi-locus sequence type
MSH: mannose-sensitive haemagglutinin
NCBI: National Center for Biotechnology Information
PCA: principal component analysis
SNP: single nucleotide polymorphism
STs: sequence types
TA: toxin–antitoxin
T4aP: type IVa pilus
T2SS: type II secretion system
T3SS: type III secretion system
T4SS: type IV secretion system
T5SS: type V secretion system
T6SS: type VI secretion system
VFDB: virulence factor database
